# The Role of p66Shc in Diabetes: A Comprehensive Review from Bench to Bedside

**DOI:** 10.1155/2022/7703520

**Published:** 2022-11-24

**Authors:** SeyedehFatemeh Mousavi, Mohammad Amin Khazeei Tabari, Alireza Bagheri, Noosha Samieefar, Negar Shaterian, Roya Kelishadi

**Affiliations:** ^1^Student Research Committee, School of Medicine, Shahid Beheshti University of Medical Sciences, Tehran, Iran; ^2^USERN Office, Shahid Beheshti University of Medical Sciences, Tehran, Iran; ^3^Student Research Committee, Mazandaran University of Medical Sciences, Mazandaran, Iran; ^4^USERN Office, Mazandaran University of Medical Sciences, Mazandaran, Iran; ^5^Department of Genetics, Faculty of Basic Sciences, Shahrekord University, Shahrekord, Iran; ^6^Student Research Committee, School of Medicine, Jahrom University of Medical Sciences, Jahrom, Iran; ^7^USERN Office, Jahrom University of Medical Sciences, Jahrom, Iran; ^8^Child Growth and Development Research Center, Research Institute for Primordial Prevention of Non-Communicable Disease, Isfahan University of Medical Sciences, Isfahan, Iran; ^9^USERN Office, Research Institute for Primordial Prevention of Non-Communicable Disease, Isfahan University of Medical Sciences, Isfahan, Iran

## Abstract

It is well-documented that diabetes is an inflammatory and oxidative disease, with an escalating global burden. Still, there is no definite treatment for diabetes or even prevention of its harmful complications. Therefore, understanding the molecular pathways associated with diabetes might help in finding a solution. p66Shc is a member of Shc family proteins, and it is considered as an oxidative stress sensor and regulator in cells. There are inconsistent data about the role of p66Shc in inducing diabetes, but accumulating evidence supports its role in the pathogenesis of diabetes-related complications, including macro and microangiopathies. There is growing hope that by understanding and targeting molecular pathways involved in this network, prevention of diabetes or its complications would be achievable. This review provides an overview about the role of p66Shc in the development of diabetes and its complications.

## 1. Introduction

Diabetes mellitus (DM) is a global health problem at individual and public levels. In recent years, it has been identified as an inflammatory and oxidative stress-related disorder [1–3]. In fact, oxidative stress and inflammation are related to each other and to pathogenesis and prognosis of diseases such as obesity and diabetes [1, 2, 4–6]. Recent evidence suggests that increase in the production of reactive oxygen species (ROS) could lead to type 1 (T1D) and 2 (T2D) diabetes mellitus [4, 7, 8]. In addition, ROS production increases in diabetes and also in the setting of hyperglycemia in healthy individuals. Increase in ROS production, at least in part, causes the destructive effects of hyperglycemia [7, 8].

p66Shc is a member of Shc protein family. These are adaptor proteins with the ability to recruit various signaling molecules via their different domains; as a result, they have a role in several cellular pathways, including cell survival, growth, proliferation, and differentiation. p66Shc is distinct from other members based on its structure and function. p66Shc has major roles in regulating reactive oxygen species and cellular apoptosis [9]. The interaction of p66Shc and ROS is also associated with the development of diabetic pathologies [10]. Understanding the molecular pathways of oxidative stress can be one of the most important steps in prevention and treatment of diabetes and its complication [4]. The aim of this review is to provide an overview about the role of p66Shc in the development of diabetes and its complications.

## 2. What Is p66Shc?

p66Shc belongs to Shc family, the signature of this family is the common Src homology 2 domain (SH2) at C-terminal, the phosphotyrosine binding PTB domain at N-terminal, and the collagen homology 1 (CH1) at the center. This family consists of four main members: ShcA (Shc1/Shc), ShcB (Shc2/Sli), ShcC (Shc3/Rai), and ShcD (Shc4/RaLP). Proteins having Src homology 2 domain are at the center of attention, as they participate in signal transduction pathways, including growth factor receptor cascade.

ShcA was discovered in 1992 while searching for proteins containing SH2 domain. Unlike other members of this family, ShcA is expressed in a wide range of mammalian cell lines, and it is the most well-known in this family. ShcA has three isoforms which arise from two different m-RNAs of a single genetic locus and are named based on their molecular weight as p66Shc, p52Shc, and p46Shc. Besides common domains talked about earlier, p66Shc, the longest isoform of ShcA, has one extra collagen homology 2 (CH2) domain at its N-terminal. This extra domain contains the serine phosphorylation (Ser36 and Ser54) sites [4, 9, 11–15]. p66Shc also has a cytochrome c-binding (CB) site in the CH-2/PTB domains. Due to its structure, p66Shc acts differently from other isoforms. p66Shc has major roles in regulating reactive oxygen species and cellular apoptosis. Although p52Shc and p45Shc are expressed in nearly every cell type, p66Shc expression varies a lot [4, 15–17]. One point worth mentioning here is that p66Shc pathway is much more complicated than previously thought; at least in some types of cells and some situations, p66Shc can show antioxidant and antiapoptotic features [7, 18, 19].

## 3. ROS Production in Diabetes Mellitus

The amount of ROS production increases in both types of diabetes. On the other hand, evidence suggests that increase in ROS level has an important role in pathogenesis of type 1 and type 2 diabetes [4, 7]. The sources of ROS production in diabetes are mostly in mitochondrial electron chain and the NADPH oxidase pathway [7, 20, 21]. Advanced glycation end products (AGEs) which increase in diabetes are another important source of ROS production [7, 16]. Xanthine oxidase (XO) has been also introduced as another origin of ROS production in diabetic mice [22]. However, there are controversial findings in this regard. In one study in streptozotocin-induced diabetic rats, excessive mitochondrial ROS production, at least via complex I and III, were under question [7]. Another study in pancreatic beta cells of metabolically responsive rat showed that the effect of glucose on ROS level depends on its concentration. In their study, in glucose concentrations between 0 and 5 mg, ROS production was reduced, and glucose concentration up to 20 mg did not increase ROS production and even caused an increase in NADPH and FADH [2]. Based on their observation, high-glucose metabolism reduced ROS accumulation through increasing NADH which is known as an ROS scavenger [7, 23].

ROS mediates elevation in the amount of some of the inflammatory markers that can cause insulin resistance in type 2 diabetes and disease progression. One pathway that exemplifies the relationship between oxidative stress and inflammation is that increase in ROS level causes upregulation of nuclear factor *κ*B (NF-*κ*B), which is a main inflammatory modulator [1, 7, 24]. Another example is that TNF-alpha participates in p66Shc phosphorylation, and thus increases ROS level [4].

## 4. The Molecular Role of P66Shc in Diabetes

A close relationship between p66Shc and oxidative-related diseases such as diabetes was reported in several experiments [5]. It has been suggested that p66Shc can act as a novel biomarker for determining development and progression of chronic age-related diseases, neoplasms, and metabolic diseases including diabetes. There is increasing hope for using p66Shc as a therapeutic target in the future [17]. It has been demonstrated that p66Shc expression is significantly higher in at least some cell types of diabetic patients, including peripheral mononuclear cells, renal cortex, and aorta. Similarly, increase in expression of p66Shc was seen in serum and placenta of patients with gestational diabetes mellitus [4, 25–29]. Also, high glucose and diabetes mellitus can increase p66Shc expression [4, 30]. However, there are controversial data as well. For example, in one study in prediabetic patients, mild oxidative stress was noted, but interestingly, p66Shc expression was significantly decreased in prediabetic stage [1]. Another finding was that in a limited in vitro study on blastocysts, p66Shc expression did not change in high glucose concentration environments [18, 31].

Gene expression can be regulated at several stages. Histone methyltransferase, deacetylase, and histone acetyl transferase like GCN5 can affect p66Shc expression. In fact, decrease in methylation and increase in acetylation will enhance p66Shc expression [29]. It has been observed that hyperglycemia can be “memorize” in at least some types of cells such as stem cells via epigenetic changes, and once the change occurs, normoglycemia cannot undo these pathways [7, 32, 33]. However, some data suggest antioxidant agents can fade away this memory [29]. Also, C peptide shows inhibitory effects against persistent upregulation of p66Shc after glucose normalization [34].

To better understand the p66Shc roles in cellular pathways related to diabetes, some of the most important cellular pathways with p66Shc participation are mentioned.

Mitogen-activated protein kinase (MAPK) cascades are important signaling pathways which participate in a wide spectrum of cellular functions, such as proliferation, differentiation, apoptosis, and stress responses. At least four MAPK are characterized: extracellular signal-regulated kinase 1/2 (ERK 1/2), Jun kinase (JNK), p38, and ERK5. Upstream molecules of this pathways are also important. The Ras/Raf/MAPKK(MEK)/ERK pathway is the most important among all MAPK family pathways [35]. EGF, IGF-1, and T-cell antigen receptor pathway can trigger Ras/MAP kinase cascade. Shc family takes part in these cascades. For instance, these adaptor proteins form a complex with growth factor receptor-bound protein 2 (Grb2) and son of sevenless (Sos) protein, although all of Shc isoforms are target of receptor tyrosine kinases and they can be tyrosine phosphorylated after growth factor stimulation, the following consequences are not the same with different Shc family members. [4]. p66Shc has inhibitory effects on these pathways. In epidermal growth factor receptor (EGFR) pathway, when p52Shc and p46Shc bind to Grb2, they cause activation of mitogen-activated protein (MAP) kinase cascade and the c-fos (a protooncogene) promoter [29]. The downregulation of p66shc can result in persistent activation of Ras and Erk1/2 pathways [4, 18].

Several stimuli including ROS and insulin can induce expression and serine phosphorylation of p66Shc [4, 7]. Serine phosphorylation of p66Shc is important for its oxidative damage and its preapoptotic effect. In different cells and different stimulants, Erk1/2, stress-activated kinases JNK, and p38 are among the proteins in charge of Ser36 phosphorylation of p66Shc [4, 5].

Lys81 acetylation of p66Shc occurs in the setting of oxidative stimuli such as hyperglycemia and Lys81 acetylation of p66Shc facilitates S36 phosphorylation of p66Shc.

SIRT 1 is a family member of sirtuin NAD+ dependent class III histone deacetylase. SIRT 1 is known as an oxidative stress protector, also it shows antidiabetic effects [36]. Inhibition of SIRT1 causes increase in lysine acetylation of its target proteins, including histones and nonhistone proteins such as p66Shc, p53, FOXO, and e-NOS, and may contribute to the pathogenesis of T2D [8, 37]. Serine phosphorylation of p66Shc helps its recognition by prolyl isomerase 1 (PIN1). PIN1 can do cis-trans*-*isomerization of p66Shc; this pathway ultimately leads to translocation of p66Shc to mitochondria, where it will bound to mitochondrial heat shock protein 70 (mtHsp70) and TIM-TOM, in intermitochondrial space. Nevertheless, before p66Shc translocates to mitochondria, it will be dephosphorylated again. In response to cellular challenges, p66Shc-mtHSP70 inhibitory complex will disband and lead to ROS production, oxidizing cytochrome *c* and openning the permeability transition pore (MPTP) and the following consequences. Nonetheless, an experiment that used VEGF instead of hyperglycemia as their oxidative stimulant did not confirm the requirement of lysine 81 acetylation for S36 phosphorylation of p66Shc [5, 8, 18, 29, 33]. In conclusion, SIRT 1 constitutes a major effect on regulating p66Shc through direct downregulation of p66shc activity, reducing epigenetic expression of p66Shc, and downregulating p53 activity in diabetic setting [24, 38, 39]. However, hyperglycemia can directly downregulate SIRT1 deacetylation activity [15]. On the other hand, acetylated p66Shc might also downregulate SIRT1 by several pathways, for example via upregulation of microRNA-34 (miR-34a) [29, 40]. Another protein involved in this track is ubiquitin-specific peptidase 22 (USP22), which mediates SIRT1 stability and its expression. USP22 is downregulated in response to high-glucose states in INS-1 or human 1.1b4 pancreatic beta cells [37]. It has been also discovered that p38 MAPK reduces USP22 expression in HeLa cells [37]. As mentioned, c-Jun N-terminal kinase (JNK) can phosphorylate p66Shc. It has been revealed that p66Shc activation is linked to Rac1-mediated activation of NADPH oxidase. Activation of NADPH oxidase is also related to JNK activation, and overactivation of JNK would result in SIRT 1 degradation [4, 29]. Also, Rac 1 phosphorylates p66Shc at Ser54 and Thr386 that prevents p66Shc from binding to ubiquitin and therefore prevents p66Shc degradation [29].

Tumor suppressor p53 participates in oxidative stress apoptosis [38]. p53 acts differently in response to different kinds, amounts, and durations of oxidative stress. In low to moderate levels of oxidative stress, p53 activates specific pathways in order to promote cell repair. However, if P53 oppose with higher levels of stress, it will induce apoptosis [41]. p53 expression can be affected by the amount of ROS production via p66Shc. Interestingly, p66shc is a downstream effector of p53, and it is necessary for ROS-induced p53-dependant apoptosis. It seems like p66Shc takes part in p53-related cytochrome *c* release [4, 29, 42]. It is probable that p53 has some effects on p66shc expression [4, 29]. Both p53 and SIRT1 are responsible in oxidative stress-related conditions, and they are modulated in diabetic settings. In monocytes of diabetic patients, a rise in p53 accompanied by decrease in SIRT 1 was seen [38]. High-glucose states can upregulate p53, and p53 possibly via p66Shc can promote miR-34a. As mentioned, miR-34a has an inhibitory effect on SIRT1. A positive feedback loop between p53 and miR-34a is possible in endothelial cells in hyperglycemic setting. On the other hand, SIRT1 can also inhibit p53 [40]. Therefore, SIRT1 and p53 can control p66Shc expression [8, 15, 29].

It is possible that protein kinase C*δ* (PKC*δ*) participates in phosphorylation and mitochondrial translocation of p66Shc [7, 43]. High glucose and insulin via protein kinase C*δ* (PKC*δ*) induce p66Shc expression and phosphorylation in HK-2 cells, and this process is dependent on time and concentration of environmental glucose. In addition, increase in ROS can result in PKC activation [24]. PKC*δ* is from the PKC family and has important functions in several cellular pathways, including cell multiplication and cell death [44]. PKC inhibitors can downregulate NF-*κ*B activation in endothelial cells [24]. Another protein from the PKC family that is activated in high-glucose settings in HK2 cell is PKC*β* which is associated with elevated proinflammatory factors such as iNOS, IL6, TNF-*α*, and ET_A_ and can also contribute to serine phosphorylation of p66Shc and activation of the p66Shc-NADPH oxidase pathway [5, 12, 29, 45]. Moreover, p66Shc itself activates PKC*β* and causes positive feedback [8]. Recent studies show that instead of serine 36 phosphorylation, other sites of p66Shc like Ser139, Ser213, and Thr206 might be involved in association with PKC*β* [18].

Mammalian Target of Rapamycin (mTOR) cascade mediates different cellular functions including cell cycle progression, protein synthesis, and mitochondrial function. S6K protein is one of the most important effectors of mTOR pathway. High nutrients and chronic hyperinsulinemia can overactivate S6K cascade [46]. Overactivation of S6K/mTOR pathway contributes to several diseases such as diabetes and obesity [6, 47]. p66Shc can stimulate S6K and also forms a complex with S6K and IRS-1 [6, 18]. S6K activation can result in serine phosphorylation and degradation of one of the most important insulin transducers, IRS-1, and thus, downregulation of insulin responsiveness (inhibitory feedback) [6, 46]. In obese wild-type mice, insulin desensitization occurs following the overactivation of S6K and further IRS-1 degradation; here, p66Shc mostly acts as an adaptor protein rather than its redox activity. Another possible pathway is that high-energy substrates can lead to ROS production in mitochondria, and the increased level of ROS can stimulate p66Shc and thus, activation of S6K/mTOR cascade occurs. In other words, deletion of p66Shc can mimic nutrient restriction in these cells [6]. One study in normal settings showed that p66Shc has a positive role in insulin pathway through its ROS dependent activity, but this study was not accounted for context of nutrient overload and insulin resistance, and it is possible that p66Shc induces insulin response in normal settings [46].

Protein kinase B (PKB) or Akt participates in many cellular functions like cell metabolism, growth, proliferation, and survival. Phosphoinositide-3-kinase (PI3K) has a role in activation of PKB [48]. High glucose and ROS through p66Shc dependent manner stimulate the PI3K/Akt pathway. p66Shc via activation of PKB/Akt can cause activation of mTOR [6]. Activation of Akt results in phosphorylation and thus, inhibition of FKHRL1 (FOXO3a). FOXO3a is a transcription factor that belongs to O subclass of the forkhead family. FOXO3a is considered a regulatory factor for longevity and cancer. FOXO3a affects the transcription of ROS-scavenging enzymes such as catalase and superoxide dismutase and also the release of proapoptotic proteins. It has also been reported that effect of FOXO3a depends on cell types and also gene-dosing manner as too high or too low level of FOXO3a might cause cell death [4, 7, 16, 38]. Putting these data together, p66Shc reduces antioxidant level in cells [4, 5, 7, 29]. Also, PI3K/Akt axis activation leads to activation of NADPH oxidase. On the other hand, high ROS level has inhibitory effects on PI3K/Akt axis through inhibitory feedback. In another study, IGF-I mediates PI3K/Akt pathway activation and can prevent apoptosis in vascular smooth muscles; however, hyperglycemia, via p66Shc overexpression, inhibits IGF-I and mediates PI3K/Akt activation [49]. In summary, in the setting of hyperglycemia, Akt contributes to ROS-mediated pathway in both positive and negative aspects [7]. Moreover, high oxidative stress and diabetes cause accumulation in AGEs. AGEs can induce oxidative stress and inflammation, and it is possible that p66Shc participates in this pathway. AGE rises serine phosphorylation of p66Shc and thus, leads to inactivation of FOXO3a and impairment of PI3 kinase and Akt activation [29, 49]. In conclusion, p66Shc participates in elevating ROS level via increase ROS production in mitochondria by transferring electrons from cytochrome *c* to oxygen, NADPH oxidase pathway, and AGEs and also by reducing ROS scavengers. Thus, it can contribute to obesity and diabetes [7–9, 18, 29, 45].

## 5. Effect of p66shc on Glucose Transport, Metabolism, and Hemostasis

In L6 skeletal muscle cell line, cytoskeletal rearrangement in response to growth factor stimulation depends on Shc family, mostly by p46 and p52 Shc isoforms. Cytoskeletal arrangement is important for cellular shape, motility, and transferring different objects in cytoplasm, including glucose transport. p66Shc has an inhibitory role in ERK pathway, and it may act as an inhibitory feedback for other Shc isoforms in this pathway. Also, ERK activity is related to cellular movement. Cells with reduction in p66shc level and thus, overfunction of MEK/MAP kinase (ERK) pathway, undergo changes to a rounded shape and complete disturbance of actin stress fibers and focal adhesions. These cells also show impaired growth, differentiation, and DNA synthesis in response to IGF-1 stimulation. In addition, overexpression of p66Shc has inhibitory effects on IGF-1-mediated cell migration. The inhibitory role of p66Shc is necessary for cytoskeletal arrangement and normal response to IGF-1. Several studies have shown that ERK activity and cytoskeletal function are necessary for glucose transport. In L6 muscle cell lines with reduced expression of p66shc, basal glucose transport rate increased significantly, notably owing to the ERK-mediated remodeling of cytoskeletal, and also due to increased expression of glucose transporter, GLUT1, and 3 in ERK-independent pathways. In contrast, overexpression of p66Shc via adenovirus caused opposite results. This observation was confirmed by using HeLa and MEFs [4, 9, 16, 18, 50]. Some other studies partially confirm the above-mentioned observations; as an example in the primary adipocytes p66Shc-negative mice, glucose uptake after insulin stimulation increased compared to wild type; but in basal level, glucose uptake was identical to wild-type phenotype. However, controversial data exist and opposite results were also observed. To explain this finding, in a recent study, the authors suggested that these controversial results can be explained by the possibility that p66Shc has positive and negative roles in insulin pathway through participation in different points of mTOR/S6K pathway [18]. Also, controversial data exist in literatures about the effect of p66Shc and its deletion in glucose metabolism. In one study, downregulation of key glycolytic pathway enzymes like hexokinase, phosphofructokinase-1, and pyruvate kinase, and thus reduction in glycolytic capacity, was seen in the skeletal muscles of p66Shc-negative mice [18, 51]. However, in another study on MEF cells, it was demonstrated that p66Shc deletion would result in increased glycolysis and anabolism. Another experiments on HeLa and MEFs also reported that deletion of p66Shc reduces oxygen capacity and consumption and causes switching to glycolysis and anaerobic metabolism. According to their report, mTOR pathway is responsible for switching metabolism pathways toward anabolism, and p66Shc has negative effects on this axis. Increased phosphorylation of proteins participating in mTOR pathway, including S6K and Akt, was noted in p66Shc-negative cells compared to control HeLa cells [5, 18, 52].

## 6. Effect on Beta-Cell Apoptosis

A reduction in pancreatic beta cells results in onset and progression of diabetes [53]. Cells with deletion of p66Shc show increased resistance to apoptosis in contrast to diffrent external stimuli [4]. Chronic hyperglycemia causes diminution of antioxidant level in the *β*-cells and therefore, influences beta-cell function and T2D prognosis. In insulinoma, INS-1 cells of rats and human beta cells (1.1B4) and high-glucose settings induce acetylation of peroxiredoxin-3 (PRDX3) which is a mitochondrial protein with antioxidant function and acts as ROS scavengers. Acetylation of PRDX3 potentiates its hyperoxidation and results in SIRT 1 degredation. SIRT1 deficiency leads to activation of NOX-JNK-p66Shc signalosome and results in pancreatic beta-cell apoptosis. On the other hand, SIRT1, through SRT3 (one of the most important deacetylators), prevents PRDX3 acetylation [37]. In recent studies, Teneligliptin (one of drugs from DPP-4 inhibitors), has been promising for reversing these pathways by stabilizing SIRT1 and preventing beta-cell apoptosis [7, 37].

Peroxisome proliferator-activated receptor (PPAR) family is nuclear receptors and has a significant modulatory role in metabolism. PPAR-*γ*, a member of this family, demonstrates the ability to increase glucose metabolism and improves insulin sensitization [54]. In pancreatic beta cells, high glucose and inflammation, by activation of cyclin-dependent kinase 5 (CDK5), cause inhibition of PPAR-*γ*, induce ER stress, and also have inhibitory effects on SIRT1. ER stress activates Rac1-GTP-NADPH oxidase axis. Together, these pathways lead to overactivation of p66Shc followed by mitochondrial dysfunction and beta-cell Bax- (Bcl-2-associated X protein-) mediated apoptosis. Therefore, ER stress-induced apoptosis occurs by participation of CDK5-p66Shc signalosome and Bcl-2 family. Mcl-1 is an antioxidant protein which has an inhibitory effect on this pathway. It has been shown that inflammatory pathways, palmitrate, and thapsigargin downregulate Mcl-1 in *β*-cells. Myricetin might have protective effects against pancreatic beta-cell apoptosis via inhibiting CDK5 and stabilizing Mcl-1 [55].

Moreover, p66Shc is associated with pancreatic beta-cell dysfunction and apoptosis that occur in some other conditions, including lipotoxicity and ceramide (N-acetyl-sphingosine) signaling [56, 57].

## 7. Effects of p66Shc on Insulin Secretion

The abnormal Src signaling pathway has some effects on beta-cell dysfunction in T2D [55].

Increasing ROS level suppresses first phase of glucose-induced insulin secretion (GIIS) from pancreatic beta cells [7, 58–60]. However, it has been demonstrated that mitochondrial ROS (mROS) is necessary for GIIS and production of H2O2 by glucose metabolism and can act as a signal for insulin secretion [7, 60, 61]. In fact, the level of oxidative stress is important as high oxidative stress can suppress insulin secretion [7, 61, 62].

## 8. Effects of p66Shc on Insulin Sensitivity

Shc adaptor proteins including p66Shc participate in insulin receptor signaling pathways [18, 63].

ROS can induce insulin resistance via several cellular pathways [6, 7, 64]. In fact, ROS can affect insulin signaling pathways in two directions; in moderate amount of ROS level, it upregulates insulin pathway, but high amounts of ROS have destructive effects [7]. In addition, hyperglycemia, due to uneven differentiation of common myeloid progenitors (CMPs), induces myelopoiesis and leads to generation of proinflammatory cells. p66Shc is associated with these changes and thus, can result in spreading of inflammation through the body and increasing insulin resistance [2].

p66Shc is a suppressor of insulin signaling pathway. In some studies, p66Shc-deficient mice with high-fat diet showed better insulin sensitivity and better glucose tolerant compared to wild-type mice [46]. Novel p66Shc knockout mice strain (ShcL) has shown more sensitivity to insulin, like old p66Shc knockout strain (ShcP), but there are differences between these two strains. In ShcL strain, the expression of other Shc isoforms remains intact, and these mice are more susceptible to obesity induced by fatty diet. Accordingly, they are fatter but their fat cells were more sensitive to insulin compared to ShcP strain. In ShcP, we have four-fold overexpression of p46Shc that might promote lean phenotype of this group and their resistance to obesity induced by fatty diet. However, this group are less insulin-sensitive compared to ShcL strain. However, this difference was not supported by further experiments [18, 65].

p66Shc also causes insulin resistant, independent of hyperglycemia. It has been shown that overexpression of p66Shc in response to lipotoxicity and overabundance of body fat causes beta-cell dysfunction and increases insulin resistance [66]. Deletion of p66Shc in leptin-deficient mice rescues insulin response and endothelial function by IRS-1/Akt/e-NOS axis [67]. Moreover, SIRT1 is related to pathogenesis of hepatic steatosis in obesity [37], and liver steatosis is known as one of the major insulin resistance causes. Of note is that lower liver steatosis was seen in p66ko mice [6, 7].

Obesity can lead to insulin resistance and progression of diabetes. It has been discovered that at least some parts of insulin resistance induced by obesity were caused by p66Shc and mTOR pathways [6, 46, 68]. There are some controversial data in the literatures about p66Shc effect on obesity and body weight. Some studies have shown that ablation of p66Shc has protective effects against obesity and causes better insulin sensitivity; some reported that p66Shc-negative mice are leaner, but they are insulin resistant like wild-type mice [18, 69–71]. In some other studies, the body weight of knockout mice was reported equal to the wild type. In addition, ShcL mice were susceptible to high-fat-diet-induced obesity. Therefore, the role of p66Shc mechanism in regulation of body weight is far from being fully understood [18, 65].

In one study, deletion of p66Shc in leptin-deficient mice (model of type 2 diabetes) showed; although the weight of these mice are similar to p66Shc sufficient group, they were more insulin-sensitive and were significantly protected against diabetes [6]. In another experiment, insulin sensitivity and glucose tolerance were compared among p66Shc and leptin knockout mice from mixed genetic background. Mice with double deletion of p66Shc and leptin were glucose intolerant like leptin knockout mice, but the double knockout mice showed a lesser degree of glucose intolerance and better insulin sensitivity. Also, the result of p66Shc-negative mice was similar to that of wild-type lean mice, and there was no difference in FBS, GTT, and insulin tolerance tests (ITTs) [18]. In another experiment, insulin sensitivity and glucose tolerance between p66Shc knockout and wild-type mice were compared in different ages; the result of this study showed that 3-month-old p66Shc-negative mice have better glucose tolerance and insulin sensitivity compared to wild type at the same age, but at 24 months, only insulin sensitivity was improved in p66Shc-negative mice [18]. In other reports, ShcL mice and ShcP mice have shown more sensitivity to insulin compared to wild types, and this effect was maintained in the ShcP mice in spite of high-fat diet but was lost in liver of ShcL strain after high-fat diet. Contradictory data was seen in a study on p66Shc-negative lean mice and WT lean mice of C57Bl/6J genetic background. They reported that at 18 weeks old, the p66Shc-negative lean mice showed more glucose intolerance, and also at age of 30 weeks, they showed worse insulin sensitivity. Also, they compared the results of these two groups with obese p66Shc-negative mice, and the latter group showed the same degree of glucose intolerance but more insulin resistance. In a study in human adipose tissue, this finding from animal studies was supported through the observation that the deletion of p66Shc can result in lower BMI, but cannot improve or prevent diabetes [18].

**Figure 1:**
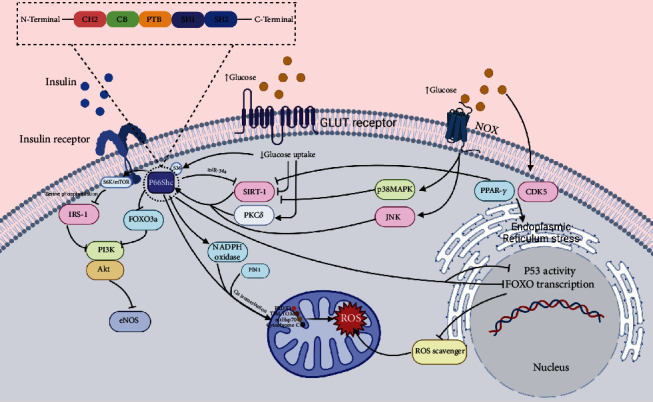
A summary of molecular pathways related to p66Shc and diabetes.

Some studies also reported p66Shc-negative mice were not resistant to the induction of diabetes mellitus via streptozotocin (a drug that induces diabetes and mimics type 1 diabetes). After injection of streptozotocin, both sp66Shc-negative and wild-type mice showed the same increase in blood glucose, but there were differences in the following consequences, such as endothelial dysfunction and renal injury [72, 73]. A summary of these molecular pathways is shown in Figure 1.

## 9. Effects of p66Shc on Life Span

It is likely that p66Shc controls the ROS level in baseline situations as well as cellular stress states, and it is possibly one of the main proteins responsible for accumulation of mitochondrial DNA damage [5]. It has been reported that p66Shc knockout mice, similar to mice under nutrients restriction, had an increase in their life span, and they could tolerate more cellular challenges [30, 42, 74, 75]. However, in a study with a higher number of mice and three different mice strain, p66Shc mice did not have longer life spans [18]. Another study on centenarian humans highlights the point that p66Shc effect on life span is more complicated than what we previously thought [18, 76]. It is suggested that p66Shc does not truly increase life span, but it can affect health span [18].

## 10. Effects of p66Shc on Diabetes-Related Complications

### 10.1. Cardiovascular Complications

Cardiovascular complications are the most common cause of mortality and morbidity in diabetic patients [77]. It was reported that P66Shc overexpression is at least partially responsible for diabetes-related cardiomyopathy and vascular dysfunction [20]. Hyperglycemia can be memorized by cells via epigenetic changes [78, 79]. Epigenetic changes in p66Shc promoter explain the cardiomyopathy even with intensive glycemic control [21]. Epigenetic changes in p66Shc promoter are associated with persistent vascular dysfunction in type 2 diabetes. Although control of HbA1C can reduce diabetic complications [80, 81], effects of glucose fluctuations cannot be reversed just by intensive HbA1C control; hence, reducing glycemic variability is an important point for alleviating diabetic cardiovascular complications [78]. Another study also confirmed this theory, by demonstrating that intensive glycemic control in long-standing poor control diabetes failed to prevent cardiovascular complications. However, in new-onset diabetes, it can reduce microvascular complications, myocardial infarction, and mortality. However, even with early treatment of hyperglycemia, episodes of elevated glucose level in this group can lead to future complications [82]. In addition to elevated glucose level, lipotoxicity is also involved in mitochondrial ROS production in diabetes. Increase in ROS level leads to activation of several pathways including increasing AGE and activation of PKC; these pathways result in more ROS production and are also associated with diabetic cardiomyopathy. ROS production is related to cardiomyocyte loss in streptomycin-induced diabetic mice and diabetic patients [20]. Some data suggest we can prevent or at least alleviate the diabetic vasculopathy via p66Shc regulation [24]. In some experiments, antioxidant strategies demonstrated the ability to prevent and to some degree reverse diabetic cardiac myopathy [83]. However, according to some studies, primary clinical results of these substances in regard to diabetic vascular complications have been disappointing so far [84]. Moreover, foam cell formation is one of the mechanisms involved in atherosclerosis [85]. Increase in ROS level via NF-*κ*B causes upregulation of inflammatory adhesion molecules and further foam cell formation [24].

In diabetic patients, p66Shc, through its ability in increasing oxidative stress, can impair myocyte and cardiac progenitor cell (CPC) viability, growth, and differentiation, thus can result in myocyte apoptosis, heart cells senescence, and eventually heart failure. p66Shc deletion can almost completely reverse these changes and maintain cardiac geometry and thus cardiac function. According to one study, p66Shc is not involved in coronary artery disease [5, 7, 12, 86]. On the other hand, in another study, epigenetic changes of p66Shc promoter with resultant overexpression of p66Shc were seen in patients with coronary artery disease [24]. In a cohort study that followed up diabetic patients for 5 to 6 years, the level of p66Shc expression in peripheral blood mononuclear cells in the beginning of the cohort did not differ based on the presence of previous complications. During the period of follow-up, the level of p66Shc was not related to diabetes complications; howbeit, patients with higher levels of p66Shc at the beginning of the cohort were more likely to develop new-onset macroangiopathy. This study concluded p66Shc can act as a predictor for likelihood of future complications [87].

Angiotensin II induces ROS production via activation of NADPH oxidases and also by activating PKC-*β*–p66Shc axis. Inhibition of renin-angiotensin-aldosterone-system (RAAS) causes a reduction in ROS level [10, 20]. Another study also confirmed PKC*β*_2_/p66Shc axis participation in diabetic cardiomyopathy; and according to their report, carvedilol, by inhibiting production of ROS and inflammatory molecules, shows protective effects against myocardial injury [88]. Also, adiponectin shows protective effects against high-glucose-induced myocardial injury, at least in part by inhibition of p66Shc [89].

Diabetes causes increase in p66Shc expression in vasculature, and p66Shc has been involved in endothelial dysfunction, atherosclerosis, and vascular hyperglycemic memory in diabetic mice. It seems lysine 81 acetylation of p66Shc has a definite role in this regard, and SIRT 1 can protect p66Shc adverse effects on vasculature. Additionally, p66Shc causes overexpression of miR-34a. miR-34a is responsible for endothelial-dependent vasorelaxation impairment. SIRT 1 is one of the most important targets of miR-34a, as saving SIRT1 can return endothelial-dependent vasorelaxation [8, 39, 40, 90]. Endothelial cells have an innate ability to regulate blood vessel diameter through producing NO and endothelium-derived relaxing factor (EDRF). In an experiment, p66Shc-negative mice were protected against diabetic vascular dysfunction. According to some reports, p66Shc-negative mice are not different in the amount of NO synthesis and cholesterol level compared to wild-type mice, but they have lower levels of peroxynitrite (ONOO, which is produced from the reaction between H2O2 and NO), nitrotyrosine, and lipid peroxidation and higher levels of NO bioavailability. However, some experiments on p66-negative mice reported elevated NO synthesis and increased expression of heme oxygenase 1, which is an antioxidant enzyme [5, 12, 16, 72].

One of the most important complications of diabetes is peripheral arterial disease (PAD) that is distinguished via imperfect traffic of hematopoietic stem/progenitor cells (HSPCs). CD4 is a marker that is used for recognizing HSPCs [91]. Hyperglycemia can be memorized by these cells and cause persistent dysfunction [32]. In one study, it has been shown that hematopoietic deletion of p66Shc in diabetic mice is enough for improving HSPC mobilization and restoring blood flow after ischemia [63]. Albiero et al. showed oncostatin M (OSM)-p66Shc pathway is responsible for diabetic-associated myelopoiesis and mobilization of hematopoietic stem/progenitor cells (HSPCs) [2], and according to this study, both hematopoietic and nonhematopoietic deletion of p66Shc is required for HSPC mobilization [2, 92]. Pioglitazone via PPAR-*γ* activation can disconnect diabetic-induced myelopoiesis from HSPC mobilopathy [92]. Another study showed that PPAR-*α* can activate phosphoinositide- (PI-) 3 kinase (PI3K)/Akt and NO axis and shows protective effects against ischemia and reperfusion injury in myocardium of type 2 diabetic rat [93]. Wils et al. reported that diabetes promotes endothelial progenitor cell (EPC) dysfunction and reduces the number of these cells which originate from HSPCs. These cells have certain roles in angiogenesis and vascular repair after ischemia and injury. Hyperglycemia and insulin resistance, through several pathways including p53/SIRT1/p66Shc axis and NO pathway, drive EPC dysregulation, and thus increases the risk of cardiovascular complications [38].

In addition, p66Shc can downregulate expression of Kruppel-like factor 2 (KLF2), which is a transcriptional factor that is crucial for endothelial function. KLF2 increases thrombomodulin (a vasoprotective agent) gene expression *[*29*]*. p66Shc participates in endothelin1 signaling pathway; this pathway ends up in cell proliferation via an ERK-dependent manner as well [29].

Some substances show a hope toward alleviating diabetic complications, for example, PKC inhibitors can improve insulin sensitivity and attenuate diabetic vascular complications based on a study on endothelial cells of diabetic patients [12, 24]. In addition, C peptide shows a protective effect against diabetic complications; for example, in high glucose settings, it reduces endothelial cell apoptosis and improves vascular injury [34, 94]. Besides, it was shown that vitamin D receptor agonist can improve vascular dysfunction via inhibition of PIN1-p66Shc axis [95]. Inhibition of PIN1 prevents vascular injury via increase in NO bioavailability, suppression of NF-*κ*B activity, and also inhibition of p66Shc-dependent ROS production and its further consequences [96].

One study suggested that p66Shc can act differently based on specific tissue and stimulants. This suggestion arose from the observation that p66Shc do not prevent heart muscle hypertrophy induced by *α*1-adrenergic, and also after endothelin1 stimulation, p66Shc via ERK-dependent manner induces cell proliferation [29].

### 10.2. Diabetic Nephropathy

Diabetes is one of the most important causes of end stage renal diseases. It has been shown that p66Shc expression increases in renal tissue of patients with diabetic nephropathy, and epigenetic changes in its promoter were observed in patients with diabetic nephropathy [27]. p66Shc-negative diabetic mice show significantly better renal function and structure compared to wild-type mice. Most of the indicators of renal function such as proteinuria and glomerular sclerosis index showed better results in the first group [4, 5, 97]. Several studies were done in order to understand p66Shc effects on diabetic nephropathy. p66Shc can promote renal tubular injury. Changes in mitochondrial dynamics have an important role in this regard. p66Shc overexpression in diabetic settings shows apoptosis in renal proximal tubule cell. p66Shc expression in PBMs might act as an indicator of renal injury [15, 98, 99]. Probucol epigenetically downregulates p66Shc expression and can prevent diabetes-induced renal tubular injury in mice [28, 94]. Some data suggest that abnormal p66Shc pathways, via affecting kidney development, and nephrogenesis can potentiate diabetic nephropathy [100]. Moreover, AGEs are involved in diabetic nephropathy [101]. In glomerular cells of wild-type diabetic mice, the rate of apoptosis was increased compared to p66Shc-negative mice [12, 97]. AGE, by p66Shc-dependent manner, induces glomerulopathy [5]. p66Shc overexpression induces K_ATP_ channel activity, which is related to increase in afferent arteriole diameter and glomerular hyperfiltration injury in diabetic rats [73]. In diabetic settings, p66Shc promotes mesangial cell injury and apoptosis. These cells preserve glomeruli's structure and function and mediate functions of filter barriers [97, 102], and piperazine ferulate (PF) can inhibit p66Shc [102]. In addition, TRPC channels are important for mesangial function. In elevated glucose states, the activity and expression of TRPC6 channel in mesangial cells decrease, but at the same time the expression of this channel in the podocytes increases. It is possible that overexpression of p66Shc is responsible for this inhibitory effect by reducing mesangial cell number, and it is likely that there is another mechanism for its effect. p66Shc also affects proliferation of mesangial cells [28]. Moreover, podocyte injury is one of the most important mechanisms of diabetic nephropathy. p66Shc overexpression decreases podocyte autophagy and induces podocytes apoptosis via notch-PTEN-PI3K/Akt/mTOR pathway [103]. Klotho is an antiaging and kidney-protective substance which is mostly secreted from renal tissue. It seems klotho has inhibitory effects on PKC*α*/p66SHC pathway, but diabetic nephropathy downregulates its secretion [104].

Studies also suggest some substance that can target p66Shc pathway; in one experiment, treatment with enzastaurin, which inhibits PKC*β*, can reduce diabetic nephropathy through inhibition of PKC*β*-p66Shc-NADPH oxidase pathway [45]. In another study, activation of PKC*δ*/p66Shc pathway was associated with tubulointerstitial injury and rottlerin production, which are PKC*δ* inhibitors, and can prevent the above-mentioned pathway [99]. Furthermore, according to one study on rats, corcumin can inhibit PKC*β*II/p66Shc axis and show nephroprotective effects [105]. Moreover, *Dioscorea zingiberensis* shows protective effects against diabetic nephropathy, and *Dioscorea zingiberensis* possibly inhibits p66Shc activity in diabetic mice with high-fat regime, therefore, resulting in reducing oxidative stress and inflammation [106]. Along with, it was shown that activated protein C (aPC) inhibits p66Shc overexpression through epigenetic changes in p66Shc promoter in diabetic rats [10, 107].

### 10.3. Retinopathy

According to one experiment, while the expression of p66Shc in the retina of diabetic and normal mice is dominantly seen in retinal ganglion cell layer and inner nuclear layer, the p66Shc expression and cellular apoptosis are significantly higher in diabetic group and increase with progression of diabetes [108]. It has been shown that in human retinal endothelial cells, high glucose increases p66Shc expression, possibly via increase in acetylated histone 3 lysine 9 (H3K9) levels and p53, and thus, promotes Rac1-Nox2 pathway that ends up in production of ROS and apoptosis of endothelial cell, so can contribute to development of diabetic retinopathy [109].

p66Shc overexpression contributes to retinal pigment epithelial cell injury and apoptosis; Exendin-4, via inhibition of c-Jun N-terminal kinase, protein kinase-*β,* and p66Shc, prevents diabetic retinopathy [110].

### 10.4. Wound Healing

Various causes lead to impaired wound healing in diabetes. Deletion of p66Shc improves the speed of wound healing via rises in collagen, granulation tissue, and bone marrow-derived angiogenic cell (BMAC) function and can improve ischemia [30, 111]. Also, Ganoderma lucidum polysaccharide (Gl-PS) improves diabetic wound healing in STZ-induced diabetic mice, and p66Shc inhibition is one of its mechanism [112].

### 10.5. Neurologic Disorders

Type 1 and type 2 diabetes make diabetic patients susceptible to cognitive disorders, especially Alzheimer's disease, and diabetic-induced oxidative stress has a significant role in this pathway, which is at least in part, independent of known amyloid accumulation mechanisms of Alzheimer's disease. Additionally, increase in ROS level can cause neuronal apoptosis, and one study demonstrated that amyloid-peptide (A) accumulation leads to phosphorylation of p66Shc by JNK [5].

Diabetes facilitates brain senescence possibly via inducing oxidative stress and inflammation through p66Shc and NF-*κ*B pathway. p66Shc-negative diabetic mice show less oxidative stress and proinflammatory markers. Furthermore, they have normal microglial cell number and activity compared to wild-type diabetic mice. As a result, fewer cognitive impairments were noted [113, 114].

Diabetic autonomic neuropathy in bone marrow occurs with reduction in SNS fiber numbers through p66Shc-dependent manner, and this pathway is one of the mechanisms involved in impairment of stem cell mobilization. Mobilization of these cells is important for vascular healing, angiogenesis, and thus after ischemic reperfusion. It has been shown that activation of beta-adrenergic receptors can increase SIRT1 expression and diabetes-induced neuropathy like chemically-induced neuropathy and decreases SIRT1 expression. p66Shc and SIRT 1 have a central role in defects in the ability of stem cell mobilization [33].

### 10.6. Erectile Dysfunction

Oxidative stress has significant role in erectile dysfunction pathogenesis [115]. p66Shc overactivity in diabetic rats leads to vascular impairment of the cavernosal tissue, and in one study, argirein showed protective effects against this pathway [116]. Moreover, melatonin through increasing SIRT1 expression shows protective effects against hyperglycemia-induced oxidative stress and erectile dysfunction [117].

### 10.7. Diabetic Embryopathy

It has been shown that hyperglycemia in pregnancy, via overactivation of JNK1/2 and p66Shc, causes mice embryonic cell apoptosis and leads to malformation [118]. However, in another study on mouse preimplantation embryo culture, exposure to high glucose level was unable to change p66Shc level, in contrast to oxygen [31].

### 10.8. Diabetes-Associated Osteoporosis

Diabetes causes dysfunction of skeleton organ. Diabetes can impair bone formation and increases the risk of bone fracture via different mechanisms [119, 120]. Diabetes causes osteoblasts apoptosis, impairs differentiation of these cells, and also promotes osteoclast differentiation both in in vivo and also in vitro if cells encounter the combination of glucose, free fatty acids (similar to in vivo environment of diabetes especially type 2), and SIRT 1 by suppressing via p66Shc/ROS/NF-*κ*B axis has inhibitory effects on this pathway [120] .

According to a study, ROS/MAPKs/NF-*κ*B/NLRP3 axis activation is one of the main mechanisms in diabetes-induced osteoporosis [121].

### 10.9. Hearing Loss

Diabetes is a risk factor for hearing loss, but the actual relationship between them is more complex. Diabetes-induced pathologies for example vasculopathy and neuropathy can contribute to hearing loss [122]. Hyperglycemia leads to mitochondrial dysfunction and disturbs the function of mesangial cells of stria vascularis of the inner ear, which has elevated mitochondrial numbers due to their high-energy consumption [122]. In an in vitro experiment, glucose-induced oxidative stress causes overexpression of p66Shc, further cellular injury and apoptosis in these cells [123].

**Figure 2:**
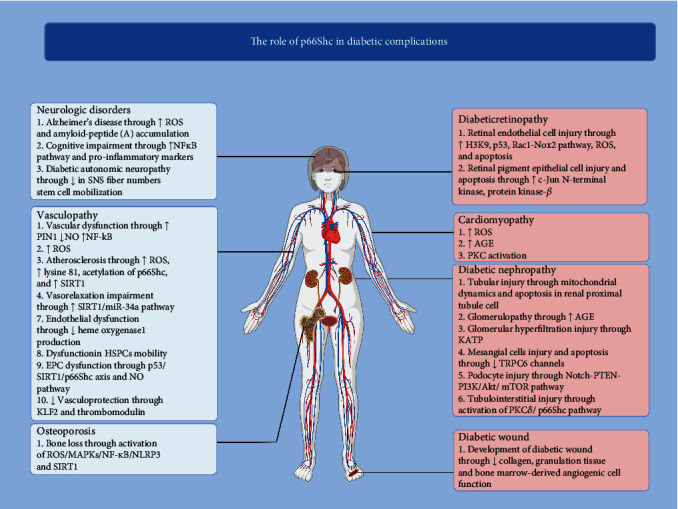
A summary of diabetic complications and the role of p66Shc.

A summary of complications and their mechanisms is available in Figure 2, and the promising drugs that could be considered as a target for treatment are summarized in Table 1.

## 11. Conclusion

The role of p66Shc in the development of diabetes and its complications remains to be determined. Studies on p66Sch-negative cells/mice demonstrated controversial results in pathways that involve in diabetes pathogenesis. By way of illustration, the results of studies on effect of p66Sch deletion on insulin sensitivity, glucose transport, and metabolisms were inconclusive. Therefore, its role in inducing diabetes is still far from being totally understood. Nevertheless, several studies confirmed the role of p66Shc in promoting diabetes-related complications, including macro- and microangiopathies. Nonetheless, the actual pathways involved with p66Shc in cells seem to be more complicated. Other roles of p66Shc in cells must be considered in the way of targeting it as a treatment strategy. Therefore, we need to know and conduct more experiments in this field, which is worthwhile for further investigation. It is also possible that future treatments will be able to target just specific functions of p66Shc protein in cells. Several medications and substances that target p66Shc or its upstream and downstream molecules have beneficial effects against diabetic-associated complications. However, more investigations are needed, since diabetes mellitus is now a global epidemic, and special attention should be paid for preventing or at least alleviating diabetes and its complications; targeting p66Shc has been promising to help us in this regard.

## Figures and Tables

**Table 1:** Substances which show benefit against diabetes-related complication. Of note, more investigations are needed, and until today, there is not enough evidence to support the actual effect of these agents.

	Substances	Effect and mechanism	Target population	Reference
Cardiovascular	Renin-angiotensin-aldosterone-system (RAAS) inhibitors	Inhibits NADPH-PKC-*β*–p66Shc axis, so decrease ROS level and cellular apoptosis	—	[20]
	Carvedilol	Reduces ROS level and inflammatory markers	Streptozotocin-induced diabetic rat	[88]
	Adiponectin	Prevents myocardial injury by inhibition of p66Shc	High glucose-treated human cardiac myocytes	[89]
	Pioglitazone	Partially preserves HSPC mobilization by PPAR-*γ* activation and downregulation of OSM and p66Shc	In diabetic rats and diabetic patients	[92]
	PKC inhibitors like ruboxistaurin and GF109203X	Decrease diabetes-related cardiovascular complication and rescue endothelial vasodilation through reducing NADPH-related ROS production and modulating p66Shc	Diabetic patients	[12, 24]
	C peptide	Inhibits persistent overactivation of p66Shc and p53 after glucose normalization, reduces ONOO(-) and ROS production, decreases endothelial cell apoptosis, and improves vascular injury	In vitro and in vivo in human umbilical vein endothelial cells and diabetic mice	[34, 94]
	Vitamin D receptor agonist	Inhibits PIN1-p66Shc axis and improves vascular dysfunction	Streptozotocin-induced diabetic mice	[95]
Diabetic nephropathy	RAAS inhibitors	Since angiotensin II upregulates p66Shc in proximal tubule cells, RAAS inhibitors can have protective effect	—	[10]
	Probucol	Epigenetically downregulates p66Shc expression and can prevent diabetes-induced renal tubular injury	Streptozotocin-induced diabetic mice and high glucose-treated HK-2 cells	[28, 94]
	Piperazine ferulate (PF)	Can inhibit p66Shc and has protective effect against high glucose-induced mesangial injury	In vitro mesangial cells and in vivo on diabetic mice	[102]
	Enzastaurin	Inhibits PKC*β*-p66shc-NADPH oxidase pathway and has protective effect against diabetic nephropathy	Streptozotocin-induced diabetic rats and in vitro on high glucose-treated human renal proximal tubule epithelial cells (HK-2 cells)	[45]
	Rottlerin	A PKC*δ* inhibitor, thus has inhibitory effect on PKC*δ*/p66Shc axis	High glucose-treated HK-2 cells	[99]
	Corcumin	Inhibits PKC*β*II/p66Shc axis and prevents diabetic nephropathy	Streptozotocin-induced diabetic rats	[105]
	Dioscorea zingiberensis	Possibly inhibits p66Shc activity. It shows protective effects against diabetic-induced nephropathy.	High-fat diet/streptozotocin-induced diabetic mice	[106]
	Activated protein C (aPC)	Causes epigenetic changes in p66Shc promoter and suppresses its expression	Rats with diabetic nephropathy	[10, 107].
	Resveratrol	Activates SIRT1, thus suppresses p66Shc and improves mitochondrial function	Glucose-treated mesangial cells and	[10]
Retinopathy	Exendin-4	By inhibition of JNK, protein kinase-*β,* and p66Shc, it has protective effect against diabetic retinopathy	Adult human retinal pigment epithelial-19 cells	[110]
Wound healing	Ganoderma lucidum polysaccharide (Gl-PS)	Improves diabetic wound healing and increases angiogenesis. p66Shc suppression is one of its mechanisms	STZ-induced diabetic mice	[112]
Erectile dysfunction	Argirein	Inhibiton of p66Shc overactivity	Diabetic rats	[116]
